# Identifying Dynamic Memory Effects on Vegetation State Using Recurrent Neural Networks

**DOI:** 10.3389/fdata.2019.00031

**Published:** 2019-10-23

**Authors:** Basil Kraft, Martin Jung, Marco Körner, Christian Requena Mesa, José Cortés, Markus Reichstein

**Affiliations:** ^1^Department of Biogeochemical Integration, Max Planck Institute for Biogeochemistry, Jena, Germany; ^2^Department of Aerospace and Geodesy, Technical University of Munich, Munich, Germany; ^3^German Aerospace Center (DLR), Institute of Data Science, Jena, Germany; ^4^Department of Computer Science, Friedrich Schiller University, Jena, Germany; ^5^Department of Geography, Friedrich Schiller University, Jena, Germany

**Keywords:** memory effects, lag effects, recurrent neural network (RNN), long short-term memory (LSTM) network, normalized difference vegetation index (NDVI)

## Abstract

Vegetation state is largely driven by climate and the complexity of involved processes leads to non-linear interactions over multiple time-scales. Recently, the role of temporally lagged dependencies, so-called memory effects, has been emphasized and studied using data-driven methods, relying on a vast amount of Earth observation and climate data. However, the employed models are often not able to represent the highly non-linear processes and do not represent time explicitly. Thus, data-driven study of vegetation dynamics demands new approaches that are able to model complex sequences. The success of Recurrent Neural Networks (RNNs) in other disciplines dealing with sequential data, such as Natural Language Processing, suggests adoption of this method for Earth system sciences. Here, we used a Long Short-Term Memory (LSTM) architecture to fit a global model for Normalized Difference Vegetation Index (NDVI), a proxy for vegetation state, by using climate time-series and static variables representing soil properties and land cover as predictor variables. Furthermore, a set of permutation experiments was performed with the objective to identify memory effects and to better understand the scales on which they act under different environmental conditions. This was done by comparing models that have limited access to temporal context, which was achieved through sequence permutation during model training. We performed a cross-validation with spatio-temporal blocking to deal with the auto-correlation present in the data and to increase the generalizability of the findings. With a full temporal model, global NDVI was predicted with *R*^2^ of 0.943 and *RMSE* of 0.056. The temporal model explained 14% more variance than the non-memory model on global level. The strongest differences were found in arid and semiarid regions, where the improvement was up to 25%. Our results show that memory effects matter on global scale, with the strongest effects occurring in sub-tropical and transitional water-driven biomes.

## 1. Introduction

In the past decades, terrestrial ecosystems have been recognized to play a key role in the global carbon cycle as a sink of atmospheric CO_2_, acting as a buffer for human carbon emissions (Bonan, 2015). Links between terrestrial carbon uptake to short- and midterm climate variations are still poorly understood and therefore, identifying driving mechanisms of vegetation state is crucial (Reichstein et al., 2013).

While the large-scale spatial distribution of vegetation mainly depends on climatologies, short-term dependencies of vegetation dynamics on climate variability are more complex (Papagiannopoulou et al., 2017a). This complexity expresses in dynamic interactions on multiple temporal scales, generating patterns that can only be understood and predicted considering antecedent ecosystem states and environmental conditions (Chave, 2013; De Keersmaecker et al., 2015; Seddon et al., 2016). These time-lagged impacts, so-called memory effects, have long been neglected, but have gained attention recently (Frank et al., 2015; Ogle et al., 2015).

Recently, different studies investigated memory effects to understand how vegetation reacts to climate on global level and how vulnerable ecosystems are toward weather extremes. Still, a profound comprehension of the involved processes is lacking (Ogle et al., 2015). Nevertheless, progress toward a better understanding was made. Seddon et al. (2016), for example, used an auto-regressive approach to model vegetation state as a function of temperature, water availability, cloud cover and the past vegetation state to determine sensitivity of vegetation toward and importance of the climate drivers. Similarly, De Keersmaecker et al. (2015) deployed a multiple linear regression model to analyze ecosystem resistance and resilience. They modeled anomalies of Normalized Difference Vegetation Index (NDVI), a proxy for vegetation state (Tucker, 1979), as a function of temperature anomalies, a drought index and past NDVI anomalies. Liu et al. (2018) used multiple linear regression to investigate the sensitivity of vegetation toward climate variability and to assess water memory effects. Wu et al. (2015) analyzed the impact of temperature, precipitation and solar short-wave irradiation on vegetation state, using a linear regression framework with lagged variables. In the mentioned studies, the learned model coefficients were linked to memory effects or the closely related ecosystem resilience. These studies provided important insights into memory effects, meteorological drivers of vegetation and its sensitivity toward environmental conditions. However, there is evidence that linear models are not able to adequately represent the temporal interactions inherent to ecosystem processes (Papagiannopoulou et al., 2017a). Thus, non-linear approaches that can cope with this complexity, are worthwhile exploring. To this end, Papagiannopoulou et al. (2017a) developed a Granger causality framework based on random forests to analyze the impact of climate drivers on anomalies of vegetation state and showed that non-linear approaches are needed to model vegetation dynamics. Other non-linear approaches to study global vegetation dynamics, however, have not been tested to our knowledge.

We take this opportunity to test the applicability of a state-of-the-art machine learning model to study global memory effects: Recurrent Neural Networks (RNNs). RNNs maintain a hidden state representing the system's memory (Werbos, 1990; Goodfellow et al., 2016). This memory evolves through time and is accessed for making predictions in interaction with concurrent factors. The model learns during training what share of information must be retained, forgotten and updated in order to predict the target variable and thus learns a complex representation of the modeled system. A widely used instance of the RNN model is the Long Short-Term Memory (LSTM) network (Hochreiter and Schmidhuber, 1997; Greff et al., 2017) that solves some of the shortcomings of the standard RNN. LSTMs have been proven to perform excellently on sequential data, for example in speech recognition (Graves et al., 2013), energy load forecasting (Marino et al., 2016), or crop field classification (Rußwurm and Körner, 2017, 2018). LSTMs model time explicitly and can learn interactions on multiple time-scales (Lipton et al., 2015; Reichstein et al., 2019) and can easily be extended in a modular fashion. Further, LSTMs allow the usage of raw time-series as input rather than lagged and aggregated features. For an introduction to Deep Learning and related terms we refer to Goodfellow et al. (2016), also available online (https://www.deeplearningbook.org/). For Deep Learning in the context of Earth system sciences, we recommend Reichstein et al. (2019).

In this study, we model NDVI using precipitation, temperature, short-wave irradiation and relative humidity, together with static variables representing land cover and soil properties as predictor variables. To quantify memory effects, we test and extend a time-series permutation approach that has been contemplated by Reichstein et al. (2018) and applied to CO_2_ fluxes at site level by Besnard et al. (2019). By permuting the feature and target time-series in unison during model training, the model is restricted to learn instantaneous effects only, which allows to quantify the model improvement when including memory effects. Here, we extend this method by using a block-permutation approach: By successively permuting the time-series while keeping blocks of a given length in original order during training, we limit the access to past observations of meteorological time-series to a specific length. The different models are then analyzed and compared to improve our understanding of memory effects. We consider this study a “proof of concept” that introduces a new approach for using machine learning for process understanding.

## 2. Materials and Methods

### 2.1. Vegetation Data (NDVI)

The Global Inventory Monitoring and Modeling System (GIMMS) NDVI 3g v1 (update of the NDVI 3g v0 dataset, Pinzon and Tucker, 2014) is a widely used, 15-daily global product based on data collected by the Advanced Very High Resolution Radiometer (AVHRR) that spans the period of July 1981 to December 2015. We used 33 years of the data from 1983 to 2015 (792 time-steps) in order to match the cross-validation scheme described later. To match other data used in this study and to reduce noise as well as observations gaps, the NDVI data was aggregated from its original spatial resolution of 0.083 to 0.5°. Only non-interpolated observations with good quality were used, and pixel-time-steps were dropped if more than 50% missing data was present at aggregation level. Also, aggregated pixels with more than 50% missing data in the time dimension were rejected, which mainly removes high latitude regions. Finally, pixels with more than 20% water are dropped to exclude coastal areas, and such with more than 50% barren were removed to exclude deserts. This speeds up model training while only locations with a marginal vegetation signal are removed.

### 2.2. Explanatory Variables

A total of 27 explanatory variables were used of which 6 were dynamic and 21 static. The dynamic variables 2 m air temperature (mean, minimum, and maximum), 2 m relative humidity and incoming short-wave radiation from ERA-Interim (Dee et al., 2011) and precipitation from the Multi-Source Weighted-Ensemble Precipitation (MSWEP) global precipitation dataset version 2.0 (Beck et al., 2019) were temporally aggregated to match the 15-daily NDVI data. Static variables used are Available Water Capacity from the Harmonized World Soil Database version 1.1 (FAO/IIASA/ISRIC/ISSCAS/JRC, 2009) and Water Table Depth (Fan et al., 2013, provided by the Global Water Scarcity Information Service: http://glowasis.eu). In addition, Land Cover Fractions (LCF) for the classes *Water, Evergreen Needleleaf Forest, Evergreen Broadleaf Forest, Deciduous Needleleaf Forest, Deciduous Broadleaf Forest, Mixed Forest, Closed Shrublands, Open Shrublands, Woody Savannas, Savannas, Grasslands, Permanent Wetlands, Croplands, Urban and Built-up, Cropland/Natural vegetation mosaic, Snow and ice, Barren or Sparsely Vegetated* were derived from Moderate Resolution Imaging Spectroradiometer (MODIS) MCD12Q1 collection 5 (Friedl et al., 2010). Finally, C4 fractions for the classes *Croplands* and *Croplands/Natural Vegetation* mosaic were obtained from Monfreda et al. (2008). All data was aggregated to 0.5° resolution. For an analysis of the effect of using static variables as predictors on the model performance and patterns of memory effects, we refer the reader to the Supplementary Material, section 1.

### 2.3. Modeling Approach

To model global vegetation dynamics, we chose an RNN architecture. RNNs efficiently encode information seen at past time-steps. This property emerges from its hidden state *h*, representing the memory of the network (Goodfellow et al., 2016). Information is extracted context-based from the state *h*^〈*t*−1〉^ and is used together with predictor *X*^〈*t*〉^ to compute output *h*^〈*t*〉^, which is also the input for the next time-step. An extensively reported issue with the standard RNN is the vanishing and exploding gradient problem (Pascanu et al., 2013), which limits its power to capture long-term dependencies. Thus, more complex models, such as the LSTM are used in practice to circumvent this issue (Greff et al., 2017).

**Figure 1:**
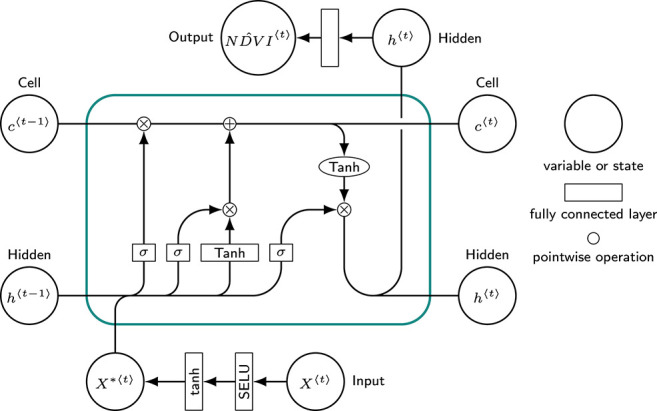
The proposed model: At its core, it consists of a standard Long Short-Term Memory (LSTM) network. In addition, the input variables *X*^〈*t*〉^ (a time-step *t* from the time-series concatenated with the static variables) are fed through two fully connected layers, yielding X^*〈*t*〉^. X^*〈*t*〉^ is concatenated to the hidden state of the last time step *h*^〈*t*−1〉^ and then passed through the LSTM's internal layers. The cell *c*^〈*t*〉^ bypasses the non-linear transformations to maintain long-term dependencies. The output *h*^〈*t*〉^ is passed through a fully connected layer to map the LSTM's output to a single value, $N\hat{D}VI$^*〈*t*〉^, the prediction for time-step *t*. Figure adapted from colah.github.io/posts/2015-08-Understanding-LSTMs.

The model architecture is illustrated in Figure 1. To find an optimal set of hyper-parameters for the model, we performed a grid search (searched range reported in brackets). The 27 predictor variables were standardized and each time-step was passed through a fully connected neural network with 2 (1–3) layers, each consisting of 128 (32–256) nodes. Dropout regularization of 0.1 (0.0–0.4) was applied after both layers. The output was used as input for a single (1–3) LSTM layer with a hidden size of 256 (32–512) nodes. A fully connected layer was attached to the output in order to map *h*^〈*t*〉^ to ${N\hat{D}VI}^{\langle t\rangle }$. We used a mini-batch size of 20 (10–100) and Adam optimizer (Kingma and Ba, 2014) with a learning rate of 0.001 (0.0001–0.1) and Mean Squared Error (MSE) as objective function. Early stopping was used as regularization to avoid over-fitting on the training data. The model was implemented in PyTorch v0.4 (Paszke et al., 2017).

### 2.4. Cross-Validation

To achieve a biased-reduced assessment of memory effects of climate variables on vegetation, we performed a *k*-fold cross-validation with spatial and temporal blocking. In a simple *k*-fold cross-validation, the data samples are randomly divided into *k* sets and each of them is used consecutively either for model training, validation or testing. Since most environmental variables are structured in space and time (Legendre, 1993), a random partitioning of the samples would possibly introduce a biased estimation of memory effects: Neglected covariates, as well as the model itself, often lead to residuals that are structured in space and time. The model can overfit the emerging residual dependency structure using predictor variables (Roberts et al., 2017) and as a consequence, we would overestimate memory effects of climate variables. Therefore, we performed a spatio-temporal cross-validation.

**Figure 2:**
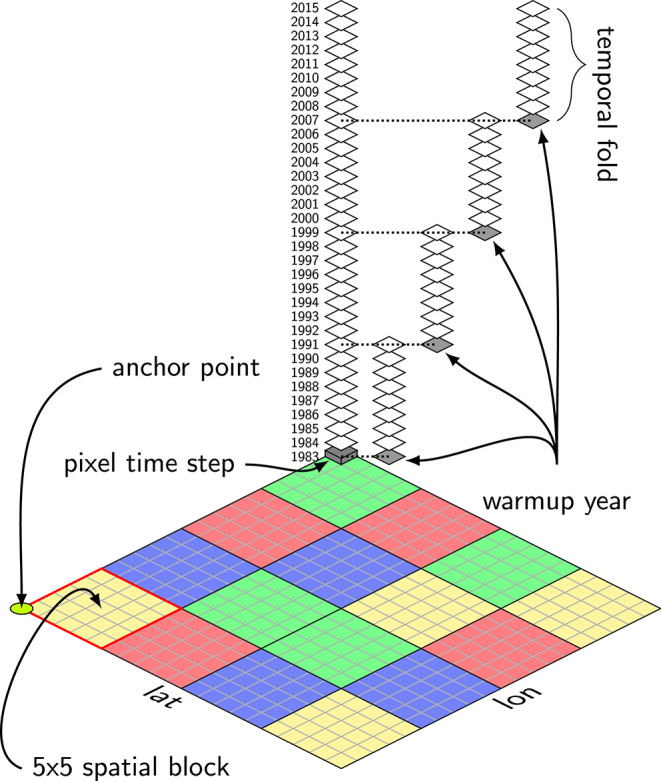
Spatio-temporal cross-validation scheme: The 4 temporal folds consist of 9 years of 15 daily consecutive data, each overlapping by 1 year, the warmup period. While the temporal partitioning is fixed, the spatial blocking is random; consecutive blocks of 5 × 5 pixels are assigned to 1 of 4 spatial folds (
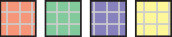
). Each color represents one spatial fold. 2 of the 4 spatial folds are used for training, 1 for validating and 1 for testing. For a given setting (e.g., training: 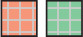, validating: 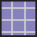, testing: 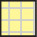), 3 of the temporal folds are used for training and the remaining temporal fold is used for validation and testing. Both the spatial and temporal folds are iterated until each pixel time-step is predicted once (in the test set). The entire cross-validation is repeated 10 times with changing anchor point (such that the points covered by one 5 × 5 block are varying) and random assignment of the blocks to one of the spatial folds.

We subdivided the spatial and temporal domain into consecutive blocks and assigned all elements of a block to one of the cross-validation sets. The choice of the block size is a trade-off between data limits, computational requirements and autocorrelation requirements (Roberts et al., 2017). Spatial blocking was done by subdividing the global raster into blocks of 5 × 5 pixels. Each 5 × 5 block was randomly assigned to one of 4 spatial folds. To account for temporal autocorrelation, the time-series were split into 4-folds of 9 years, overlapping by 1 year. The overlapping corresponds to the warmup period which is applied as the LSTM's state is initialized as zero and has to encode some of the time-series history first before becoming fully effective. The cross-validation scheme is illustrated in Figure 2.

Model training was done by iteratively using 2 spatial sets for training, 1 for validation and 1 for testing. For each of these combinations, 1 temporal block was used for validation and test while the other 3 were used for training. Note that we did not separate validation and test set in the temporal domain to not further reduce the sample size used for training, which is one of the above-mentioned trade-offs. As the model performance showed a low sensitivity toward the hyperparameters, we expect that this had a low impact on the results.

As the random assignment of the spatial blocks to the cross-validation sets may not be ideal (e.g., underrepresentation of some regions in the training set), anchor point of the spatial blocks and their assignment to the sets were varied randomly in 10 repetitions. For each of these repetitions, independent predictions for the test sets were retrieved. Each fold contained about 37% of the data for training (10,300,000 observations) and 6% for validation (1,650,000 observations). With the 4 folds from temporal, the 4 folds from spatial blocking and the 10 repetitions we ended up with 160 independent runs per model. We used the median of the 10 runs as final predictions.

### 2.5. Model Evaluation

To assess the model's predictive performance, we used the Root Mean Squared Error (*RMSE*) and the *R*^2^. We decomposed the raw time-series (NDVI_RAW_) into the median seasonal cycle (NDVI_MSC_) and the anomalies (NDVI_ANO_). NDVI_MSC_ was calculated pixel-wise as the median of the time-series across all years and NDVI_ANO_ as the difference of NDVI_RAW_ and NDVI_MSC_. The decomposition was derived individually for the observations and the predictions. To quantify global model performance and memory effects, we used robust metrics based on *R*^2^ and *RMSE*. First, we aggregated the observed and predicted time-series per hydro-climatic biome (*b*), as defined by Papagiannopoulou et al. (2018), by using the pixel-area weighted average (yielding $R_{b}^{2}$ and *RMSE*_*b*_). The biome-specific metrics were then aggregated to global level using the biome-area (*A*_*b*_) weighted mean:

$$\begin{array}{lll} R_{global}^{2} & = & \frac{1}{A}\overset{B}{\underset{b=1}{\sum }}R_{b}^{2}*A_{b}\\ RMSE_{global} & = & \frac{1}{A}\overset{B}{\underset{b=1}{\sum }}RMSE_{b}*A_{b} \end{array}$$

where *A* is the total area. This aggregation was done because NDVI_ANO_ has a low signal-to-noise ratio compared to NDVI_MSC_ and NDVI_RAW_, which has two causes: First, NDVI_ANO_ has a weaker signal (lower amplitude) than NDVI_MSC_ and NDVI_RAW_ in most cases. Second, NDVI_MSC_ was calculated as the median over several years, which lowers the impact of noise while this is not the case for NDVI_RAW_ and NDVI_ANO_. In order to compare model performance among the different decompositions, we prefer a metric that corrects for this imbalance. $R_{global}^{2}$ and *RMSE*_*global*_ reflect how large-scale NDVI patterns are reproduced while keeping the impact of data noise low.

### 2.6. Identification of Memory Effects

**Figure 3:**
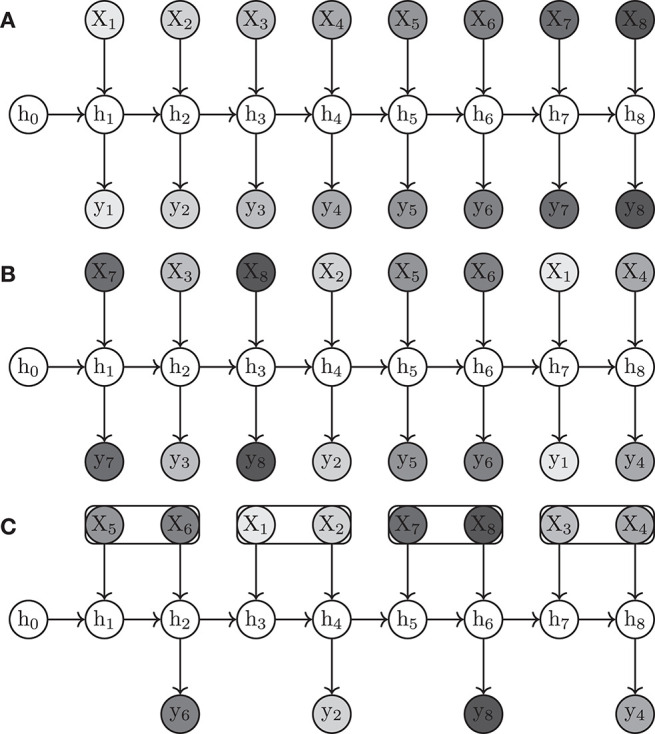
Permutation scheme for M_full_ **(A)**, M_0_ **(B)**, and M_1_ **(C)**. For model M_n_, permutation is done at each training step in order to restrict the memory *n* observations into past. The feature (*X*) and target (*y*) time-series are permuted in unison. *h*_*t*_ represents the hidden state of the model at time-step *t*. M_full_ learns the full memory effects as the time-series are not permuted, M_0_ only learns instantaneous effects, the time-series are permuted randomly. For M_1_, blocks of size 2 are permuted randomly and the first block starts at position randomly chosen from {0, …, *n*} to vary the elements covered by the blocks. During training, only the last element of each block is used in the loss calculation as *n* antecedent elements must be in original order, which is not the case for other elements.

To quantify memory effects, we trained multiple models with limited access to temporal context: During training, the dynamic features (climate variables) and the target (NDVI) time-series were permuted at each training step in unison, keeping *n* antecedent elements in original order, referred to as model M_n_ (Figure 3). Validation and prediction were done on non-permuted time-series. We use the case *n* = 1 for illustration: Here, NDVI_*t*_ is a function of *X* = {*X*_*t*−1_, *X*_*t*_}, which includes the instantaneous effect (*t* → *t*) plus one past observation (*t*−1 → *t*), hence memory of length *n* = 1, corresponding to 15 days. There are two special cases, the *full* memory model M_full_, where no permutation is done and M_0_, which is the *non-memory* model where the time-series are permuted randomly without blocking. To assess memory effects of different lengths, multiple models M_n_ with *n* = {*full*, 0, 1, 2, 3, 4, 5, 6} corresponding to {*full*, 0, 15, 30, 45, 60, 75, 90} days were computed. This choice was based on preliminary experiments, showing that the model performance was flattening after a lag of 90 days and the need to restrict the number of model runs. Note that although the permutation does destroy the order of the time-series before element *t* − *n*, the model can still learn from the distribution of the previous values.

We used the metric $Mem_{n}=R_{n}^{2}-R_{0}^{2}$ to quantify memory effects, where *n* denotes the number of antecedent observations being included. *Mem* is the difference in *R*^2^ between two models describing the impact of giving more temporal context on the model performance. For brevity, *Mem* refers to the total memory effects derived from M_full_ and M_0_.

To determine pixels of significant memory effects, we performed a permutation test. Our test statistic is the memory effect *Mem* and our null hypothesis was that *Mem* is equal to 0–meaning that on average, the models have the same performance. Each prediction can be labeled as coming from M_0_ and M_full_, and under the null hypothesis, they are exchangeable. For the permutation test, we permuted these labels 999 times (for all pixels simultaneously) and calculated each test statistic for each pixel at each permutation. The *p*-value is the proportion of test statistics that are as extreme as our observed test statistic. Since the permutation test was done on each pixel, we incurred in the multiple testing problem: As we perform thousands of simultaneous tests, it is more likely to observe significance just by chance. This was addressed by using the distribution of the maximum statistic to determine the threshold of significance at each pixel (Cortés et al. in preparation). At each permutation, we saved the maximum of the absolute value of the test statistic amongst all pixels, max(∣*Mem*∣). With the original data's maximum, these form the distribution of the maximum statistic. The threshold for significance at the pixel level was determined by the 90th percentile of this distribution.

## 3. Results

### 3.1. Model Performance

First, we take a look at the global model performance of the full memory model M_full_ and the non-memory model M_0_. Therefore, pooled—all pixels and time-steps combined—metrics *RMSE* and *R*^2^ were calculated. M_full_ achieved an *RMSE* of 0.056 compared to model M_0_ with an *RMSE* of 0.068. This is an error reduction of 14%. The *R*^2^ increased by 2.8% from 0.916 to 0.943 from M_0_ to M_full_. As the global variability of NDVI is largely caused by spatial variability (68%), we also looked at the *R*^2^ after removing the mean from each time-series. There, the improvement was 8.8% from 0.807 to 0.878.

**Figure 4:**
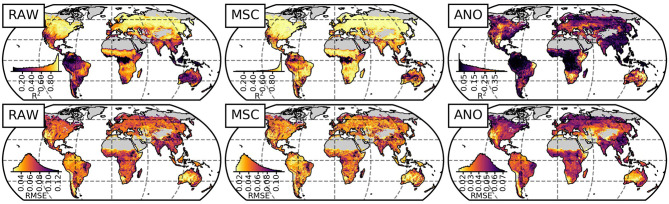
Global model performance of the full memory model M_full_ based on NDVI time-series from 1984 to 2015 in terms of *R*^2^ (top row) and *RMSE* (bottom row) for the raw time-series (RAW, left column), median seasonal cycle (MSC, center column) and anomalies (ANO, right column). The histograms are area-weighted.

The spatial variability of the model performance for M_full_ is illustrated in Figure 4. A high *R*^2^ in terms of NDVI_RAW_ and NDVI_MSC_ is achieved in the northern temperate and boreal regions, eastern South America, as well as Savanna and Steppe ecosystems of Africa—regions of distinct seasonal NDVI signal. In contrast, rainforests and dry regions, where the seasonal cycle is less pronounced, show lower values of *R*^2^, as errors take larger effects due to lower overall variance. For NDVI_ANO_, *R*^2^ is lower in general but achieves values between 0.25 and 0.4 in arid and semiarid regions. The *RMSE* of NDVI_RAW_ and NDVI_MSC_ is distributed more homogeneously, low values are found in arid regions due to the low vegetation signal.

### 3.2. Global Memory Effects

Global memory effects based on the aggregated $R_{global}^{2}$ for NDVI_RAW_, NDVI_MSC_ and NDVI_ANO_ are shown in Table 1. While M_full_ performs better in all cases, memory effects on NDVI_ANO_ are stronger than on NDVI_MSC_ in terms of absolute and relative increases of explained variance. Yet, note that for the seasonal cycle, the fraction of unexplained variance is halved from 12 to 7%, which is also reflected in the 50% decrease of the *RMSE*_*global*_ in Table 1.

**Table 1:** Model performance of models Mfull and M_0_ for NDVI_RAW_, NDVI_MSC_, and NDVI_ANO_.

		**NDVI_RAW_**	**NDVI_MSC_**	**NDVI_ANO_**
$R_{global}^{2}$	M_0_	0.848	0.881	0.323
	M_*full*_	0.904	0.928	0.465
	% increase	6.3	6.3	30.6
	*Mem*	0.06	0.06	0.14
*RMSE*_*global*_	M_0_	0.025	0.017	0.018
	M_*full*_	0.017	0.008	0.015
	% decrease	28.9	50.9	15.0

*The metrics were calculated from area-weighted, per bioclimatic region aggregated time-series*.

**Figure 5:**
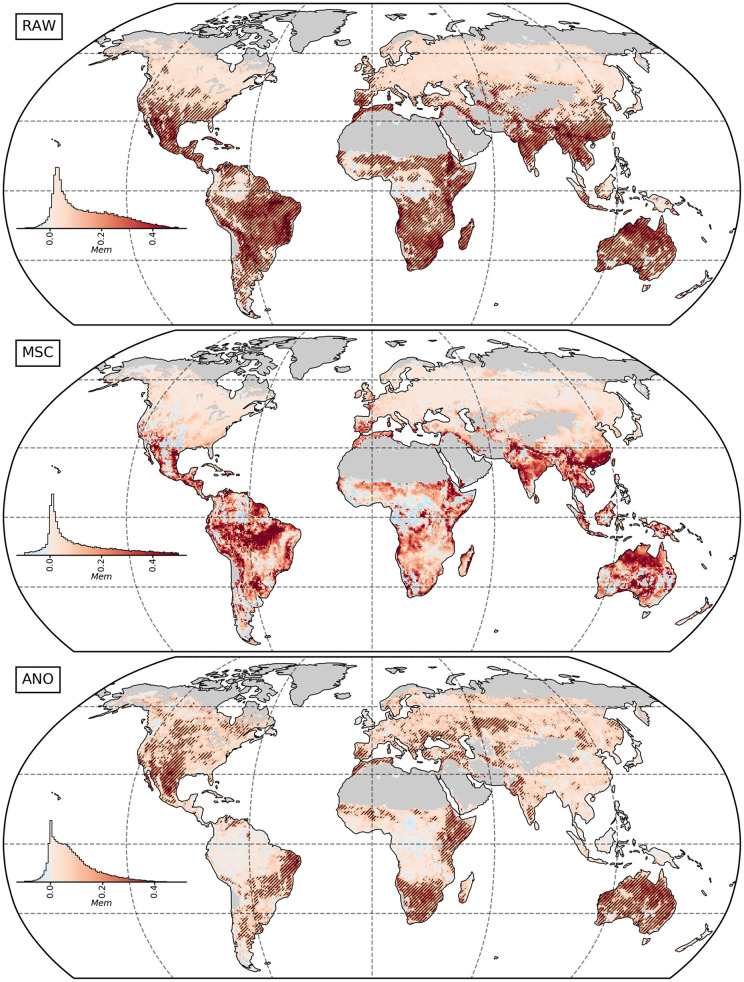
Global memory effects based on NDVI time-series from 1984 to 2015 for the raw time-series (RAW, top), median seasonal cycle (MSC, center) and anomalies (ANO, bottom). Values represent the difference in *R*^2^ between the full memory model (M_full_) and the non-memory (M_0_). Black striped areas 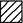 indicate significant memory effects after accounting for the multiplicity (α = 0.1). The histograms are area-weighted.

Figure 5 shows the spatial variability of memory effects. Significant effects were detected in transitional and sub-tropical biomes in general and—to a lower extent—mid-latitude water-driven climates, while the weak effects in temperate, boreal and rainforest climates were not significant on pixel basis. Accounting for antecedent climate conditions improves *R*^2^ for NDVI_MSC_ mainly in the tropical belt. However, these effects were not found to be significant. Finally, hotspots of significant memory effects for NDVI_ANO_ are similar to those of NDVI_RAW_, but more concentrated on arid and semiarid regions. Some areas show negative memory effects, especially in the case of NDVI_MSC_. Note that a small number of pixels has negative correlations, which is not reflected by the *R*^2^. However, these negative correlations are close to zero (not shown) and thus neglectable.

### 3.3. Biome-Specific Memory Effects

To understand how vegetation state is affected by antecedent climate under different environmental conditions, we take a look at biome-specific memory effects and how they change along climatic gradients.

**Figure 6:**
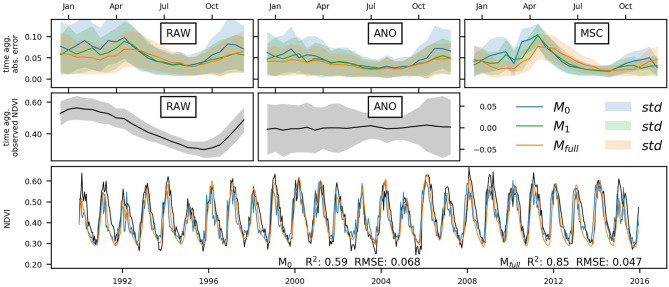
Model predictions for a 10 × 5 pixel area located in the Chobe national park, Botswana (~ 19°S 24°E). The time-aggregated absolute error and its standard deviation over all pixels of M_0_, M_1_, M_full_ are shown in the top row, time aggregated observed NDVI in middle row (note that the MSC is contained in the RAW plot), and a subset of the observed and predicted NDVI time-series from 1990 to 2015 in the bottom row.

First, we illustrate the predicted time-series for the different models with a regional example exhibiting strong memory effects (Figure 6): The Chobe National Park is located in Northern Botswana (~ 19°S 24°E) and has a transitional water-driven climate with a distinct dry and wet season, the latter starting in October and ending in April. The selected area is—compared to its surroundings—only marginally affected by wildfires (see Fox et al., 2017 for further details). Both models, M_full_ and M_0_ predict the overall patterns well, however, M_full_ performs considerably better. During low vegetation activity outside the raining season, the models perform equally with comparable variability of the error. In the rainy season when vegetation is active, the anomalies are stronger in general. Here, the full memory model M_full_ performs best, followed by M_1_. The error variation of M_0_ is larger during this period, whilst M_full_ errors have the lowest variation.

**Figure 7:**
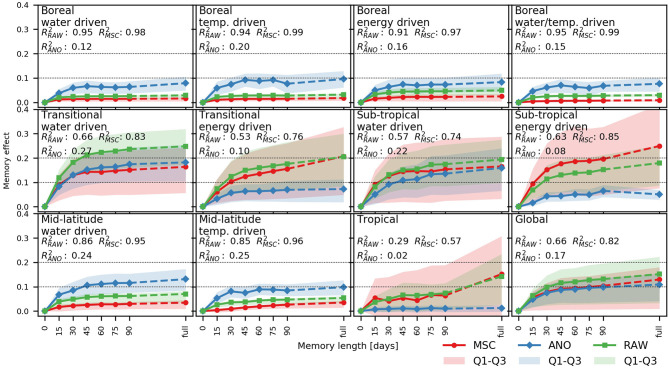
Global and biome-specific mean and interquartile range (Q1–Q3) of memory effects (*Mem*) for NDVI_RAW_ (mean removed), NDVI_MSC_ (mean removed) and NDVI_ANO_. The x axis represents the memory length, the number of days that are taken into account by the model. The shown *R*^2^ reflect the performance of the full memory model (M_full_). See Papagiannopoulou et al. (2018) for biome definition.

We further tested the impact of memory length on model performance on global as well as on biome level compared to baseline M_0_ (Figure 7), based on the permutation approach. In general, the model performance is increasing with more temporal context in a saturating way. Even if the model performance is not strictly increasing in all cases with longer memory, a positive (asymptotic) relationship was found. Some biomes show a small drop in model performance with increasing memory length. We must keep in mind that the global MSE is minimized in model training. The different models may invest in reducing MSE in different regions as long as the global cost decreases, thus we only expect the global model performance to increase strictly, while regional discrepancies are expected. On global level, memory effects on NDVI_RAW_, NDVI_MSC_, and NDVI_ANO_ are congruent. Transitional and sub-tropical biomes show strong yet highly variable memory effects on NDVI_MSC_. Distinct memory effects on NDVI_ANO_ are found in water-driven ecosystems.

**Figure 8:**
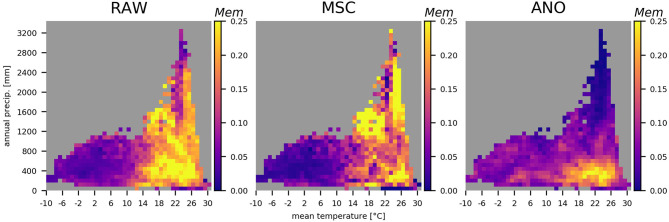
Memory effects (*Mem*) in the climate space, annual precipitation vs. temperature for NDVI_RAW_ (left), NDVI_MSC_ (middle), and NDVI_ANO_ (right). The spatial variability of NDVI_RAW_ and NDVI_MSC_ has been removed, i.e., the mean of each pixel's time-series has been subtracted prior to calculating *Mem*. Gray cells represent cases with <10 values.

Furthermore, we look at memory effects in the climate space of mean annual precipitation and temperature (Figure 8). For NDVI_RAW_, we observe increasing memory effects with higher mean temperature, similar to NDVI_MSC_. Below a threshold of around 14°C, memory effects are barely present. For NDVI_MSC_, precipitation seems to play a minor role. In contrast, memory effects on NDVI_ANO_ are higher with lower mean precipitation and higher temperatures. We see low memory effects above 700 mm annual precipitation and again, mean temperatures below 14°C.

**Figure 9:**
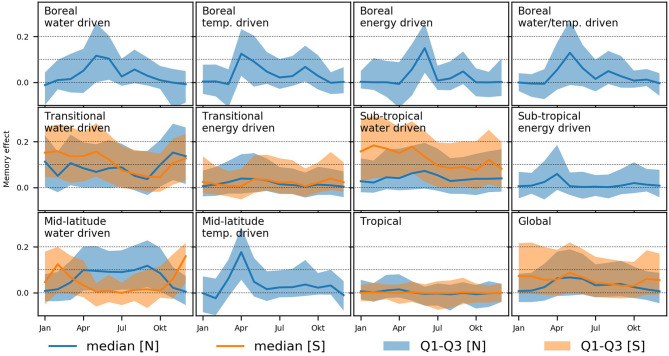
Monthly biome-specific memory effects for the Northern [N] and Southern [S] Hemisphere. The metric reflects how much better the full memory model (M_full_) explains the variance across years per month compared to the non-memory model (M_0_). Cases with less that 10 pixels are not shown. See Papagiannopoulou et al. (2018) for biome definition.

Finally, we show the inter-biome mean and variation of memory effects per month separately for the Northern and Southern Hemisphere (Figure 9). In other words, this is the increase in explained variance across years per month from the non-memory model M_0_ to the full memory M_full_ model. Note that we only display the results for NDVI_RAW_, as NDVI_ANO_ yields the same results and the approach is not applicable to NDVI_MSC_. In boreal regions, the patterns are widely consistent, with small or no memory effects in winter, stronger effects in the start of the growing season and moderate effects at peak vegetation activity with a peak toward autumn. The transitional and sub-tropical water-driven biomes exhibit stronger memory effects in the Southern Hemisphere, with high values from December to May. The respective energy-driven regions show low memory effects in general. Furthermore, we see remarkable differences between the water and temperature-driven mid-latitudes: The water-driven regions show opposite patterns in Northern and Southern hemisphere, strongest memory effects occur in summer during the growing season. In temperature-driven regions, however, we see a distinct peak in the beginning of the growing season in spring and substantially lower memory effects during the remaining time of active vegetation. The topics, finally, show no memory effects of monthly variations.

## 4. Discussion

### 4.1. Memory Effects on Vegetation State

We found memory effects on global scale with a bigger impact on the anomalies of vegetation state than on the seasonal cycle and generally lower impact on boreal and temperate climates and tropical rainforests. We detected large regional variations of memory effects and linked them to hydro-climatic biomes and climate gradients.

Our results shown in Figure 7 suggest that sub-monthly, short-term memory effects play a dominant role while the impact of mid-term memory is weaker. For temperature and energy-driven ecosystems, lower memory effects on vegetation anomalies were found, which aligns with findings by Wu et al. (2015), Seddon et al. (2016), and Papagiannopoulou et al. (2017b). In water-driven regions, except for the boreal climates, we observe strong memory effects on vegetation anomalies, which was also found by the aforementioned studies.

We found evidence that ecosystems in colder climates with a mean temperature below 10–15°C are less affected by memory effects in general (Figure 8). Above this threshold, memory effects on the median seasonal cycle of vegetation state do not depend on mean annual precipitation, whereas effects on the anomalies are stronger with an annual rainfall below 700 mm. The strongest memory effects are found in sub-tropical and transitional ecosystems (Figure 5). The effect is similar for the seasonal cycle between energy and water-driven subregions, while the anomalies are much stronger affected by past climate in respective water-driven regions.

Sub-tropical water-driven regions—containing the arid and semiarid regions of the world (Papagiannopoulou et al., 2018)—are mainly shaped through patterns of precipitation, events that often occur in short pulses, followed by dry phases of varying length (Snyder and Tartowski, 2006). Through the limited water availability, vegetation dynamics in these regions largely depend on water storage in soils. Anomalies in soil moisture can last over several months (Koster et al., 2004), potentially leading to strong memory effects. While small precipitation pulses often cannot penetrate soil layers below 20–30 cm, clustered events in interaction with lower temperatures can refill deeper soil water storage. This resource can be accessed by deeper rooted plants, some even specialize on extracting water from different soil layers through the season (Schwinning and Ehleringer, 2001). This buffering of precipitation events in soil combined with large anomalies of precipitation can lead to strong memory effects, which is reflected in our results.

Similar patterns occur in transitional water-driven ecosystems, building the transition from arid and semiarid regions to humid climates. These ecosystems are still largely limited by water availability (Papagiannopoulou et al., 2018) and exhibit a higher vegetation density than sub-tropical regions. Arid and to a lower extent semiarid ecosystem are sparsely vegetated and thus, a generally low vegetation signal is observed. The small variations in the NDVI and thus the low signal-to-noise ratio may mask effects that we try to identify. In arid and semiarid ecosystems of the Southern Hemisphere, the memory effects are occurring during active vegetation phase in the rainy season (Figure 9), which is also the case in the regional example shown in Figure 6. In the Northern Hemisphere, however, the link between precipitation and memory effect is less evident.

In the boreal and mid-latitude water-limited biomes, we see patterns of strong memory effects in spring (Figure 9). This is supposedly related to snowmelt and phenological effects of temperature. To determine when the snow cover disappeared or the top layer of the soil thawed, a certain amount of temporal context is needed, leading to relatively strong memory effects. Further, vegetation greenup timing in these ecosystems depends on the history of temperatures during previous months, which is often modeled as temperature sums in phenological models. In addition, some plants require chilling before warming effects can be effective (Migliavacca et al., 2012). Since the start of the growing season itself has a lagged impact on productivity after spring, e.g., as a consequence of more or less accumulated biomass, we see an impact of memory effects related to the spring vegetation dynamics lasting until around June. The length of memory effects (Figure 7) is similar for all boreal biomes with a maximum length of 15–30 days and stronger effects on vegetation anomalies than on the median seasonal cycle. This is counter-intuitive, as we would expect to see a strong dependency of the phenology on antecedent weather patterns due to the aforementioned cumulative temperature effects. However, the seasonal variations are well-predicted by both models (*R*^2^ > 0.95), hence we see only small memory effects, even if a large fraction of the non-explained variance of the non-memory model is explained additionally by the full memory model. Moderate memory effects are observed in the remaining growing season, we expect that an increasing drought stress in boreal regions could alter the temporal dependencies in the future (Barichivich et al., 2014).

### 4.2. Time-Series Permutation Approach

An evaluation of the presented approach is challenging because there is no ground-truth of memory effects. However, we can assess the plausibility of the results in consideration of our understanding of ecosystem processes. We looked at biome-specific monthly memory effects and showed a regional example, where the full memory model performs best and a model with shorter memory length still performs better than the non-memory model. The differences in model performance were associated with periods of active vegetation, where predictions were better and more robust when including more memory. In contrast, dry seasons with barely any vegetation activity or winter periods in boreal regions are captured equally by all models. This suggests that the found memory effects are not just an artifact but are indeed linked to vegetation dynamics. Furthermore, we looked at the length of memory effects and found that models accounting for longer temporal context perform better. The found relationships between climate gradients and memory effects align well with prior knowledge about ecosystem functioning.

Another way to evaluate the time-series permutation approach is a comparison with other studies. This turns out to be challenging as these studies (e.g., De Keersmaecker et al., 2015; Wu et al., 2015; Seddon et al., 2016; Papagiannopoulou et al., 2017b; Liu et al., 2018) use other predictor variables with different spatial and temporal resolution and different approaches (e.g., global vs. pixel-wise optimized). Due to some similarities in the study design and presentation of results, we can conduct a direct comparison to Wu et al. (2015): They employed a linear model with the lagged predictor variables temperature, precipitation and solar radiation to model monthly global NDVI on pixel basis. The authors used the regression coefficients to interpret drivers of and memory effect on vegetation state. Based on a visual inspection of the spatial model performance, our model (Figure 4) seems to perform better in terms of *R*^2^, even if trained globally and spatio-temporal cross-validation was applied (see section 4.3.2 for further discussion). The found patterns of memory effects align in general, the same major hotspots are detected, yet our results indicate more wide-spread memory effects. It is possible that the regions we detected in addition are characterized by strong non-linear climate-vegetation interactions (Foley et al., 1998; Bonan, 2015; Papagiannopoulou et al., 2017a) and cannot be represented by a linear model as a consequence.

Papagiannopoulou et al. (2017a) (and the follow-up study Papagiannopoulou et al., 2017b) take a different approach based on a non-linear Granger causality framework: They quantified the model improvement from a model that uses past NDVI anomalies only compared to a model that uses climate variables in addition. The 4,571 (3,197 in the follow-up) climate variables include lagged and cumulative features and extreme indices. In a comparison, we must keep in mind that the reported “Granger causality on vegetation” may not be directly comparable to our memory effects metrics and that the temporal resolution of the time-series differ. While—based on a visual inspection—the main patterns of memory effects on the NDVI anomalies (Papagiannopoulou et al., 2017a) seem widely congruent with our findings (Figure 4, anomalies), the most striking difference are the significantly lower effects we found in the Sahel. Interestingly, this is also the region where the LSTM model performs worse than the pixel-wise trained random forest model. These discrepancies may attribute to the different resolutions of the time-series, or to the global vs. pixel-wise modeling approach (further discussed in section 4.3.2).

A drawback of the presented permutation approach is that we cannot attribute memory effects to single variables. Yet, we linked the strongest memory effects to water-limited ecosystems, which was also found by previous studies. We can conclude that, even though results are similar, we see regional differences, and that further development and discussion of the different approaches is needed.

Another way of identifying memory effects may be to apply the permutation approach after the training. In other words, the LSTM which has learned the dynamic effects in the data will be given a permuted time-series in the prediction. This resembles the permutation approach for studying variable importance with other machine learning approaches like random forests.

### 4.3. Advantages and Limitations

#### 4.3.1. Data Limitations

Remote sensing data is inherently affected by errors related to data processing, the sensor, atmospheric effects and scene properties (Friedl et al., 2001). As a consequence, some regions—for example such with a complex topography—exhibit larger measurement errors, which affects the reliability of the results. Alike, the climatic reanalysis datasets used as predictor variables are affected by uncertainties linked to the underlying datasets and the modeling approach. A further limitation is the spatial and temporal resolution of the data. It is possible—yet not well-understood—that the temporal resolution (15 days) masks important short-term ecological processes that may propagate to longer temporal scales. Similarly, the spatial resolution of 0.5° integrates finer-grained local variations, leaving us with a smoothed signal.

Furthermore, the NDVI's dynamic range is limited since the signal saturates with dense vegetation. This poses an issue especially in dense forest areas like rainforests, where the NDVI shows little to no seasonality (Huete et al., 2006) and the anomalies mainly reflect noise. Thus, the results regarding rainforest areas should be taken with a grain of salt.

In addition, the model is limited by the choice of predictor variables: Ecosystem processes are highly complex and vegetation state depends on a vast number of factors, like nutrient availability (Fisher et al., 2012), human and natural disturbances (Reichstein et al., 2013; Trumbore et al., 2015), surface and sub-surface water flow (Koirala et al., 2017) and many others that are not included in the model. As a consequence, the interactions of the climate with those variables are neglected.

#### 4.3.2. Global Modeling Approach

While previous studies looking into memory effects or related topics (e.g., De Keersmaecker et al., 2015; Wu et al., 2015; Seddon et al., 2016; Papagiannopoulou et al., 2017a,b; Liu et al., 2018) trained a model per pixel, we used a global modeling approach: A main objective of this study was to test the applicability of LSTMs to represent global vegetation dynamics. This choice was motivated by the great success of LSTMs in many other domains: LSTMs are dynamic models that are able to capture dependencies on multiple scales and—in theory—of unlimited length. LSTMs can be applied to raw time-series opposed to approaches that work on lagged and aggregated features (Lipton et al., 2015). This renders the approach fully data-driven, as no feature design choices are necessary. Furthermore, such a model can be easily extended in a modular fashion to include spatial context using Convolutional Neural Networks, for example. In this sense, the presented approach is generic. As such models can easily have thousands of parameters, they require large amounts of data to be trained. The length of satellite observation time-series (in our case ~800 time-steps) is far away from being sufficient. With a global modeling approach, the dataset is much bigger and more adequate for a deep learning approach. Moreover, this approach achieves a unified global predictive model.

The global modeling approach was further motivated by the fact that the datasets are autocorrelated in space. We follow Roberts et al. (2017), who suggest that spatial cross-validation should be performed in all cases when dealing with environmental datasets. Especially for machine learning methods with high flexibility, overfitting is a problem that needs to be addressed. This choice, however, has a negative side-effect: The model's ability to adapt to local characteristics is limited and thus, some specificities cannot be learned. Rather, the model learns generalizable memory effects and therefore, the estimates of memory effects are conservative. In an effort to counter this issue, we included static variables that should help the model to implicitly link local differences to environmental conditions. In section 1 of the Supplementary Material, we showed that adding static variables improved model performance and made the predictions more robust. Furthermore, including these variables leads to a finer-grained picture of memory effects. This indicates that the global model learns specific local system behavior by linking it to actual local conditions rather than by “memorizing.”

A further drawback of the global modeling scope is that the model—with the objective to reduce global loss—trades off different regions: To reduce the loss, the model may invest more of its capacity in better represented areas while neglecting under-represented regions. We expect that this is also the reason for the “negative” memory effects; from a theoretical point of view, knowing more about past environmental conditions cannot result in inferior predictions. We investigated this issue in section 2 in the Supplementary Material, where the globally trained model was compared to a model optimized for a single biome only. The memory effects and length were qualitatively similar. However, the geographic distribution of the memory effects on the median seasonal cycle showed substantial differences, while the patterns for the anomalies were more congruent. Thus, we recommend interpreting the memory effects regarding the median seasonal cycle with caution. This problem could be reduced by using higher resolution data and adding covariates that reflect these local variabilities better, e.g., human factors and additional soil properties.

### 4.4. Applications

RNNs are still rarely used to model Earth observation time-series. As shown here, RNNs are well-suited to model such data, as they are able to extract complex features from raw data with the benefit of rendering feature design unnecessary. Other than for diagnostic modeling, RNNs can also be used for upscaling of fluxes, gap-filling or benchmarking of physical models, for example. The time-series permutation approach presented here can easily be applied to other fields where a profound understanding of memory effects is pivotal, such as hydrology.

### 4.5. Conclusion

In this study, we have tested the applicability of an LSTM network to model Earth system variables using multivariate predictors. We used 33 years of climate variables together with static soil and land cover features to model 15 daily satellite based NDVI observations. The model was able to learn the global spatial and temporal variability of vegetation dynamics to a satisfying degree. This demonstrates the great capabilities of LSTMs, which are still rarely used in Earth system sciences, yet their potential is known from other disciplines.

Furthermore, we used a time-series permutation approach to identify memory effects of climate on vegetation state. Our results confirm findings from previous studies and highlight some new aspects of memory effects: While the geographic distribution widely agrees with other studies, we linked memory effects to climate gradients and took a closer look at their biome-specific temporal occurrence and length. The presented approach requires minimal prior knowledge of the domain and can be combined with powerful machine learning models. These properties render the approach into a useful tool that expands existing methods, possibly serving as a benchmark for approaches being able to do a more detailed analysis of variable contributions to memory effects.

## Data Availability Statement

All datasets analyzed for this study are included in the manuscript and the Supplementary Files.

## Author Contributions

BK conducted this study in the framework of his doctoral studies with the supervision of MR, MJ, and MK, who helped to conceive and plan the experiment, as well as discussing results on a regular base. BK performed the data processing, model setup and analysis, and wrote the manuscript with the help of MR, MJ, and MK. CR helped with regular critical comments and discussions and comments on the final draft. JC performed the test for significance for the global map of memory effects.

### Conflict of Interest

The authors declare that the research was conducted in the absence of any commercial or financial relationships that could be construed as a potential conflict of interest.
