# Supplementary material for: Identifying Dynamic Memory Effects on Vegetation State Using Recurrent Neural Networks
Source: Front Big Data. 2019 Oct 23;2:31. doi: 10.3389/fdata.2019.00031 (PMC7931900; doi:10.3389/fdata.2019.00031)
Supplement: Supplementary file 1 [file Data_Sheet_1.PDF]

Supplementary Material

Identifying Dynamic Memory Effects on Vegetation State Using Recurrent Neural Networks, B. Kraft et al.
Correspondence: Basil Kraft, `bkraft@bgc-jena.mpg.de`

1 IMPACT OF STATIC VARIABLES ON MODEL RESULTS

As our model was trained globally, we included static variables (soil properties and land cover fractions) as predictors to help the model to better represent the spatial variability of vegetation state, but also to learn interactions of these variables and the climatic predictors. This static variables could eventually lead to a better representation of local memory effects. This assumption was tested by comparing the presented approach to a model that was trained without static variables.

Figure S1 shows the global performance for training and validation cross-validation sets. Using static variables does not only increase the model performance, it also increases robustness (less variation of cross-validation folds) and improves generalization (less performance decrease between training and validation sets). In most regions, the static variables helped finding stronger and more fine-grained patterns of memory effects, as Figure S2 shows. Some regions show weaker memory effects for the model with static variables. These regions are essentially tropical rainforests for the median seasonal cycle, where the model results are not trustworthy due to saturation of the NDVI signal, as discussed in the manuscript.

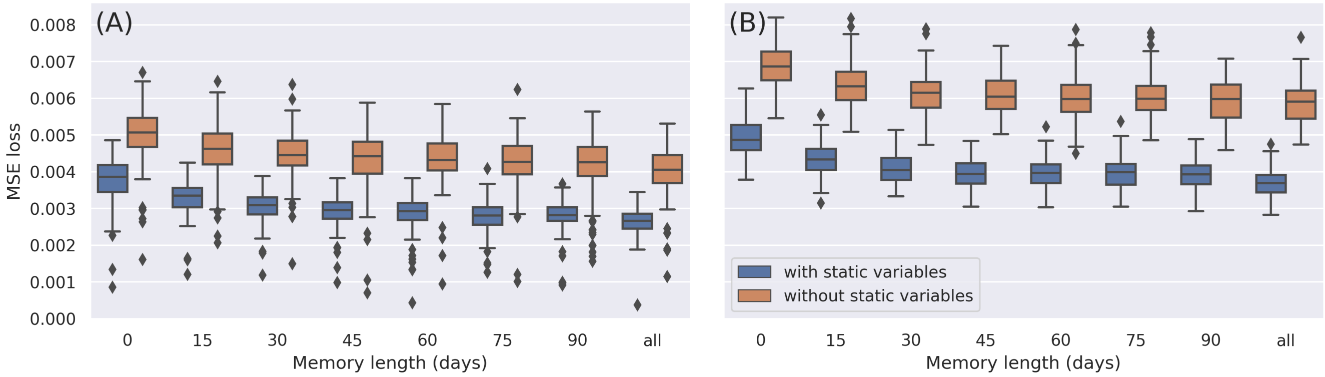

Figure S1. Global mean squared error loss for **(A)** training and **(B)** validation of the proposed model that includes static variables (soil properties and land cover fractions) versus a model that was trained without these static variables. Each box represents the model performances for the 10 repetitions of the 16 cross-validation folds (4 spatial, 4 temporal), resulting in $n = 160$ data points per box.

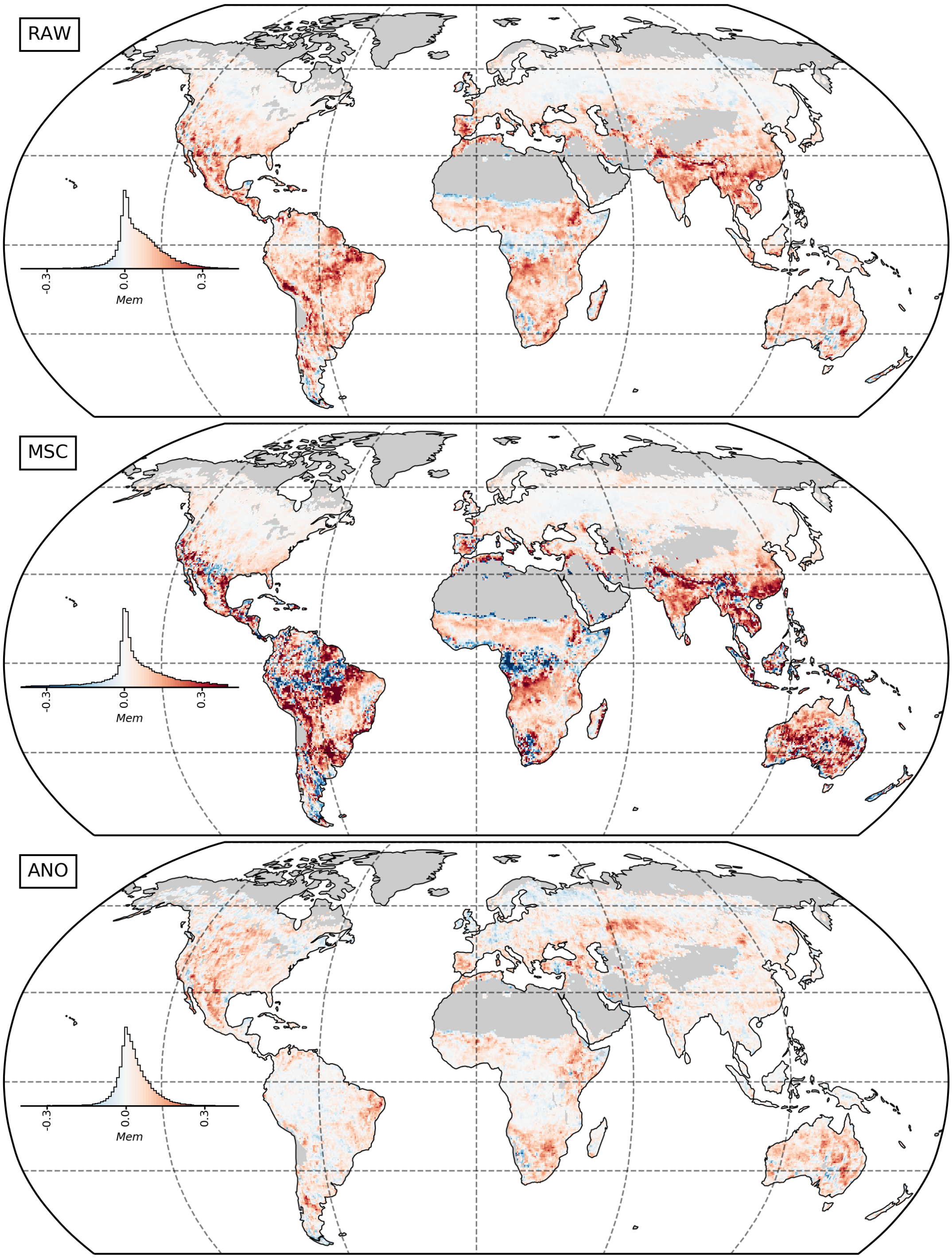

Figure S2. Differences in memory effects for the raw time-series (top), the median seasonal cycle (middle) and the anoamlies (bottom) found by the proposed model that includes stastatic variables (soil properties and land cover fractions) and a model that was trained without these static variables. Positive numbers indicate that the model that uses static variables found stronger memory effects.

2 GLOBAL VERSUS REGIONAL MODEL

To assess to what level the global modeling approach can discover local memory effects, we compared found patterns to a model that was trained exclusively on the transitional water-driven biome. This helps to understand the impact of training a global model, which has certain advantages (e.g. more training data, closer to physical processes since universal, regularization) and potential disadvantages (less adaptive to local variations).

Figure S3 shows the impact of training a global versus a regional model on the memory effects and lengths at biome level. The regional model yields slightly stronger memory effects in general, attributed primarily to the median seasonal cycle. The memory effects on anomalies are basically the same in both cases. The same conclusion can be drawn for the spatial variabilities of the differences, shown in Figure S4: The general patterns are similar, however, the patterns for the seasonal cycle show certain differences. The memory effects on the anomalies show only minor deviations.

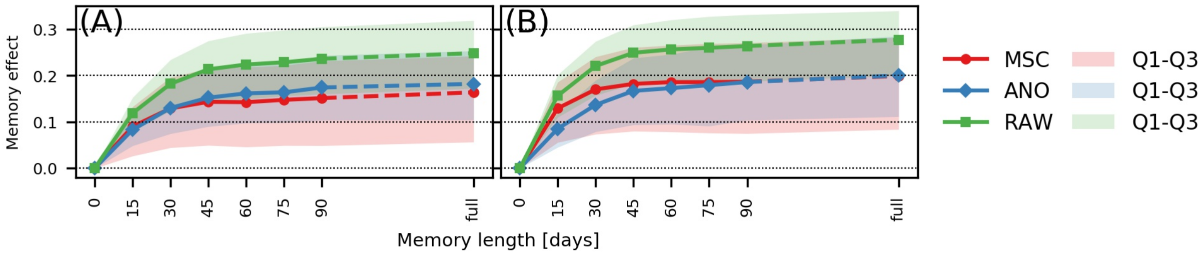

Figure S3. Memory length for the transitional water-driven biome found when the model was **(A)** trained globally versus **(B)** trained on the transitional water-driven biome only.

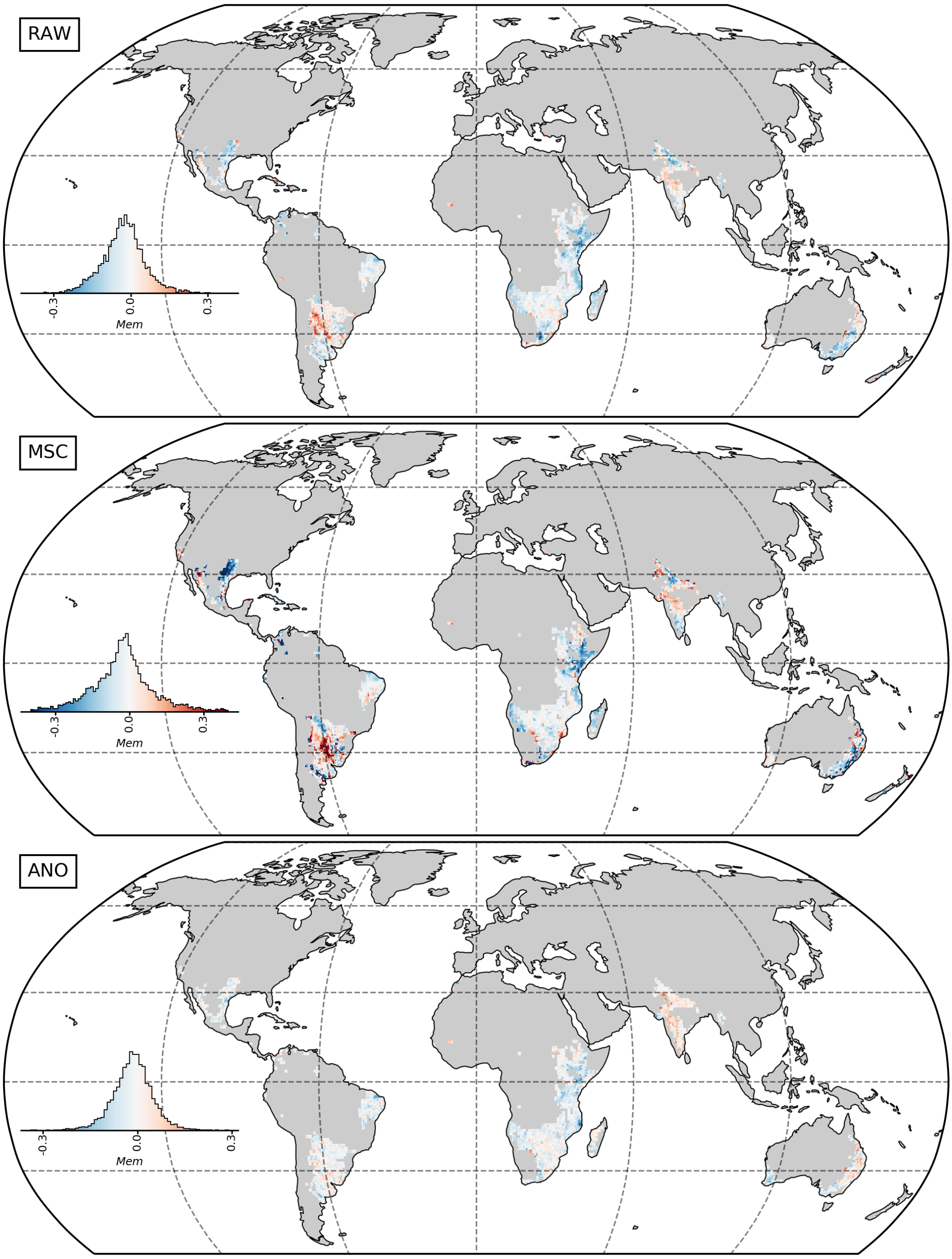

Figure S4. Differences in memory effects for the raw time-series (top), the median seasonal cycle (middle) and the anoamlies (bottom) found by the proposed model that was trained globally and a model that was trained on the transitional water-driven biome only. Positive numbers indicate that the global model found stronger memory effects than the regional model.
